# Prenatal Exposure to Tetrachloroethylene-Contaminated Drinking Water and the Risk of Adverse Birth Outcomes

**DOI:** 10.1289/ehp.10414

**Published:** 2008-02-06

**Authors:** Ann Aschengrau, Janice Weinberg, Sarah Rogers, Lisa Gallagher, Michael Winter, Veronica Vieira, Thomas Webster, David Ozonoff

**Affiliations:** 1 Department of Epidemiology; 2 Department of Biostatistics; 3 Department of Environmental Health, and; 4 Data Coordinating Center, Boston University School of Public Health, Boston, Massachusetts, USA

**Keywords:** birth outcomes, birth weight, drinking-water contamination, gestational duration, low birth weight, perchloroethylene, prematurity, tetrachloroethylene

## Abstract

**Background:**

Prior studies of prenatal exposure to tetrachloroethylene (PCE) have shown mixed results regarding its effect on birth weight and gestational age.

**Objectives:**

In this retrospective cohort study we examined whether PCE contamination of public drinking-water supplies in Massachusetts influenced the birth weight and gestational duration of children whose mothers were exposed before the child’s delivery.

**Methods:**

The study included 1,353 children whose mothers were exposed to PCE-contaminated drinking water and a comparable group of 772 children of unexposed mothers. Birth records were used to identify subjects and provide information on the outcomes. Mothers completed a questionnaire to gather information on residential histories and confounding variables. PCE exposure was estimated using EPANET water distribution system modeling software that incorporated a fate and transport model.

**Results:**

We found no meaningful associations between PCE exposure and birth weight or gestational duration. Compared with children whose mothers were unexposed during the year of the last menstrual period (LMP), adjusted mean differences in birth weight were 20.9, 6.2, 30.1, and 15.2 g for children whose mothers’ average monthly exposure during the LMP year ranged from the lowest to highest quartile. Similarly, compared with unexposed children, adjusted mean differences in gestational age were −0.2, 0.1, −0.1, and −0.2 weeks for children whose mothers’ average monthly exposure ranged from the lowest to highest quartile. Similar results were observed for two other measures of prenatal exposure.

**Conclusions:**

These results suggest that prenatal PCE exposure does not have an adverse effect on these birth outcomes at the exposure levels experienced by this population.

In 1980, the six New England states discovered that PCE (perchloroethylene, tetrachloroethylene) was leaching into drinking water from the inner vinyl lining (VL) of asbestos cement (AC) water distribution pipes. The vinyl liner, which was introduced in the late 1960s to solve taste and odor problems, had been painted onto the inner surface of the pipe in a slurry of PCE and vinyl toluene resin (Piccotex; Johns-Manville Corporation, Denver, CO). After drying for 48 hr, the pipes were shipped for installation (Demond 1982). Because PCE is a volatile solvent, it was assumed that most would evaporate by the time of pipe installation. However, more than a decade elapsed before it was discovered that large quantities of PCE remained in the liner and were slowly leaching into the public drinking-water supplies.

A substantial number of VL/AC pipes were installed in the Cape Cod region of Masssachusetts (Larsen et al. 1983). Because the lined pipe was used to replace existing pipe and to extend the water system, contamination occurred in an irregular pattern. PCE levels in residential areas of Cape Cod ranged from undetectable to 80 μg/L along main streets and from 1,600 to 7,750 μg/L on dead-end streets (Demond 1982). Because it was prohibitively expensive to replace the VL/AC pipes, a regular schedule of flushing and bleeding was instituted in the most problematic areas to reduce levels to below 40 μg/L, the suggested no response level in 1980 (Demond 1982). The current maximum contaminant level is 5 μg/L.

Animal experiments suggest an adverse effect of prenatal exposure to PCE and the closely related solvent trichloroethylene (TCE) on offspring weight and growth in several species (e.g., Elovaara et al. 1979). However, epidemiologic studies have had inconsistent results (e.g., Bove et al. 1995; Lagakos et al. 1986). We undertook this study to determine the impact of PCE-contaminated drinking water on birth weight and gestational duration using a population-based cohort of Cape Cod children.

## Materials and Methods

### Selection of study population

Children were eligible for the study if they were born 1969–1983 and their mother was living in a Cape Cod town with some VL/AC water distribution pipes at the time of their birth. Children were identified by cross-matching the maternal address on the birth certificate with data collected from water companies on the location, installation year, and diameter of all VL/AC water pipes in the Cape Cod region.

Two groups were selected: *a*) children whose mothers were exposed to PCE-contaminated drinking water before birth, and *b*) children whose mothers were unexposed before birth. A total of 1,910 children were initially designated as “exposed” based on a visual inspection of pipe distribution maps in the immediate vicinity of the maternal address. The initial exposed group included 1,862 singleton births and 24 sets of twins.

A comparison group initially designated “unexposed” was randomly selected from the remaining resident births. Unexposed children were frequency matched to exposed children on month and year of birth. The unexposed group of 1,928 children included 1,853 singleton births and 37 sets of twins or triplets. The initial exposure status of a child was considered tentative until survey data on private well use became available and more extensive exposure assessments were completed.

We reviewed birth certificates to obtain information on the names of the child and his parents; the parents’ ages and educational levels; the date of the mother’s last menstrual period; and the child’s birth weight and gestational age.

The study complied with all applicable requirements of U.S. regulations governing the use of human subjects in research. The study was approved by the institutional review boards of the Massachusetts Department of Public Health and Boston University Medical Center, and by the 24A/B/11B Review Committee at the Massachusetts Department of Public Health. All participants gave informed consent before taking part in the study.

### Follow-up and enrollment of subjects

During 2002–2003, mothers were traced to obtain their current addresses and telephone numbers. If the mother was deceased, we attempted to locate the father. Letters were sent to all traced parents requesting that they complete a self-administered questionnaire. Two follow-up letters and phone calls were made nonrespondents. As described in Table 1, enrollment patterns were similar for exposed and unexposed subjects.

We conducted analyses comparing birth certificate data on birth weight, gestational duration, and demographic characteristics among participants and nonparticipants. The mean gestational duration was similar among the two groups; however, the mean birth weight among nonparticipant children was about 100 g lighter than that of participants. Furthermore, although the race and birth year of nonparticipants were similar to those of participants, nonparticipating mothers were younger (mean age, 26.0 vs. 27.5 years), less educated (11.3% vs. 3.6% did not graduate from high school), and had more prior births (51.1% vs. 24.3% had three or more prior births) than participating mothers. These differences were present for both exposed and unexposed nonparticipants.

Questionnaires were sent to all successfully traced parents to gather information on maternal demographic characteristics; prior pregnancy outcomes; data on smoking, alcohol intake, weight gain, and complications during each pregnancy; chronic medical conditions; and other sources of solvent exposure. In addition, information was collected on the family’s residences from 1969 to the birth of the child; the proximity of residences to dry-cleaning establishments; drinking-water sources; and the mother’s water consumption and bathing habits at each dwelling. The residential history included the calendar years of residence, exact street address, and nearest cross street for all Cape Cod residences.

### Geocoding of residential addresses

All reported Cape Cod residences were incorporated into a geographic information system (GIS) by geocoding each address using ArcGIS 8.1 (ESRI, Redlands, CA). We geocoded the residences without knowledge of the exposure or birth outcome. Among the 5,324 reported addresses, 87.6% had sufficient information to be geocoded to a parcel. Another 9.6% were geocoded to the middle of the street or to its intersection with cross street because the street number was missing. The remaining 2.7 % could not be geocoded with this level of accuracy, so the 169 associated births were excluded from the analysis (Table 1).

### PCE exposure assessment

Children received initial exposure designations by a member of our research team (S.R.) who was familiar with the water distribution systems on Cape Cod. The initial designations were determined by visually inspecting maps of the pipe network in the immediate vicinity of the birth certificate address. To determine the final exposure designation, we used a leaching and transport model to estimate the mass of PCE delivered to each residence before and during the study pregnancy. The model, developed by Webler and Brown for our prior epidemiologic studies (Aschengrau et al. 2003; Webler and Brown 1993), estimates the amount of PCE entering the drinking water using the initial PCE loading in the pipe liner, the pipe’s age, and the leaching rate of PCE from the liner into the water. The leaching rate of PCE, which declines with time, was determined from laboratory experiments (Demond 1982).

The exposure assessment also requires an estimate of water flow, which is a function of the pipe configuration and number of water users. To estimate flow for the current study, we incorporated the Webler and Brown (1993) algorithm into EPANET water distribution system modeling software (www.epanet.com). Developed by the U.S. Environmental Protection Agency, this software has been applied previously in several epidemiologic investigations (e.g., Aral et al. 1996).

As a first step, we created a GIS schematic depicting the water source locations; pipe characteristics; and nodes, which are points of water consumption. Information on the locations, installation dates, and diameters of VL/AC pipes was obtained from local water departments and the Massachusetts Department of Environmental Protection (DEP; Boston, MA). The available information reflected the water system conditions around 1980.

Next, we used the schematic to assign each residence to the closest node on the distribution system. We assumed that all residences drew the same amount of water and that the water sources did not change over time. Typical values for other parameters were assumed when their variability was low or when historical data were absent.

The EPANET software incorporated these data to simulate the instantaneous flow of water through the thousands of pipe segments in each town’s network and to estimate the mass of PCE in grams delivered to subjects’ residences. We estimated three measures of prenatal PCE exposure: cumulative exposure before pregnancy, peak annual exposure before pregnancy, and average monthly exposure around the time of conception. We estimated cumulative exposure before pregnancy by summing the annual mass of PCE that entered each exposed residence from the move-in year or VL/AC pipe installation year (whichever came later) through the month and year of the last menstrual period (LMP). We were able to calculate only annual PCE exposures because we knew only the move-in and pipe installation years. We used simple percentages to estimate PCE exposure for a portion of a year. Peak exposure before pregnancy was estimated from the highest annual mass of PCE that entered an exposed residence from the move-in year or VL/AC pipe installation year (whichever came later) up to the LMP year. We estimated average monthly exposure around the time of conception by dividing the annual exposure during the LMP year by 12. We estimated the LMP from questionnaire or birth certificate data on the gestational duration and birth date. The LMP could not be estimated for 19 births, and these were excluded from the analysis (Table 1).

We estimated PCE exposure levels only for children whose mothers had complete geocoded residential histories (Table 1). Children whose mothers reported using a private well at a Cape Cod residence or who lived in a town without VL/AC pipes were assumed to have no PCE exposure during that period.

### Statistical analysis

We excluded multiple births and children born with congenital anomalies from all analyses (Table 1). The data analysis compared the birth weight and gestational duration of exposed and unexposed women. For cumulative exposure before pregnancy, we compared any exposure before the LMP month and year with no exposure before the LMP month and year. For peak annual exposure before pregnancy, we compared any exposure before the LMP year with no exposure before the LMP year. Last, for average monthly exposure, we compared any exposure during the LMP year with no exposure during the LMP year.

We used a locally weighted regression smoother (LOESS) to examine the shape of the relationship between exposure and the outcome measures (Hastie and Tibshirani 1990). These analyses did not identify any natural cut points, so we arbitrarily divided each exposure measure into quartiles.

Analyses examined birth weight and gestational age as continuous variables (in grams and weeks, respectively) and as the following dichotomous variables: low birth weight, premature birth, and intrauterine growth retardation. Low birth weight was defined as weight < 2,500 g, a premature birth was defined as gestation < 37 completed weeks, and intrauterine growth retardation was defined as a birth weight below the 10th percentile using U.S. race-, sex-, and gestational age–specific cut offs from 1970 to 1976 (Williams et al. 1982).

Crude analyses compared the means of the continuous outcomes and proportions of the categorical outcomes according to exposure. In addition, we conducted generalized estimating equation (GEE) analyses to account for non-independent outcomes arising from several children born to the same woman (Liang and Zeger 1986; Zeger and Liang 1986). Sixteen percent of the women had two or more births. The identity and logit links were used for continuous and dichotomous outcomes, respectively, while assuming equal correlation between birth outcomes from the same mother. Corresponding means, beta coefficients, and odds ratios (ORs) are reported. Ninety-five percent confidence intervals (CIs) were used to assess the statistical stability of the associations.

Covariates considered for multivariate analyses were known risk factors for low birth weight or premature delivery or non-drinking-water sources of solvent exposure. Only covariates of *a priori* interest or those associated with PCE exposure (OR ≥ 1.2 or ≤ 0.83) were controlled in the final analyses. The adjusted birth weight analyses included gestational age, maternal race, educational level, history of a low-birth-weight child, occupational exposure to solvents, use of self-service dry cleaning, and proximity of any residences to dry cleaning establishments. Adjusted birth weight analyses were also repeated without gestational age and history of low-birth-weight child.

Adjusted gestational age analyses included maternal race, educational level, prior preterm delivery, obstetric complications in the current pregnancy, occupational exposure to solvents, use of self-service dry cleaning, and the proximity of any residences to dry cleaning establishments. Adjusted gestational age analyses were also repeated without history of a preterm delivery. Analyses were also repeated with a term for drinking-water source (surface vs. ground) because of possible confounding by chlorination by-products present in one treated surface water supply.

Last, we conducted stratified analyses to determine whether there was effect measure modification by maternal age at delivery, history of prior pregnancy losses, bottled water use during pregnancy, tap water use during pregnancy, and showering habits during pregnancy.

## Results

A total of 2,125 children were available for the final analysis. According to the initial exposure designation, there were 1,077 exposed and 1,048 unexposed children. After the more refined EPANET exposure assessment, the exposure designations of 386 children changed from unexposed to exposed, and 110 changed from exposed to unexposed. Nearly all children who changed their exposure designations from unexposed to exposed had birth addresses that were further downstream from a VL/AC pipe than was originally considered exposed in the initial visual designation. The EPANET assessment of the entire distribution system indicated that these downstream locations had potential for PCE contamination. On the other hand, all children who changed their designations from exposed to unexposed had mothers who reported that the birth residence had a private well and that they did not receive any public drinking water. Thus, the final study population consisted of 1,353 subjects who were considered exposed before birth and 772 subjects who were considered unexposed before birth.

The characteristics of the exposed and unexposed groups were similar (Table 2). Mothers in both groups were predominantly white, and, on average, 27 years old when the child was born.

Comparable proportions in the exposed and unexposed groups smoked cigarettes and drank alcoholic beverages, and reported medical conditions and obstetric complications during the study pregnancy as well as non-drinking-water sources of solvent exposure. Most women had an adequate level of prenatal care.

There was wide distribution of PCE levels encompassing several orders of magnitude for all three exposure measures (Table 3). The measures were also highly correlated (pairwise Spearman correlation coefficients = 0.71–0.90, *p*-values < 0.001).

The exposure measures were based on the mass of PCE delivered to a home in each calendar year. The annual mass of PCE entering a home was diluted in an estimated 90,000 gallons of water, the annual usage of average households in Massachusetts (Massachusetts Water Resources Authority 2003), and only a small portion of this water was directly consumed by the subjects. Using this annual estimate of household water use, we converted the PCE mass delivered to a home during pregnancy to average annual point concentrations, and estimated that the PCE concentrations in the water entering the homes ranged from < 1 μg/L to 5,197 μg/L. These concentrations are consistent with actual water-sampling data from the time period (Demond 1982).

The crude and adjusted analyses showed no consistent pattern of an adverse impact on birth weight across PCE exposure levels (Tables 4 and 5). In general, birth weights of exposed infants were greater than those of unexposed infants. In addition, although the number of affected children was small, no meaningful increases were observed in the ORs for low birth weight (*n* = 52) or intrauterine growth retardation (*n* = 136) according to PCE exposure level (data not shown). There were too few children with very low birth weight to conduct meaningful analyses of this outcome.

The crude and adjusted analyses showed a consistent but small decrease in gestational duration among PCE-exposed subjects. This decrease was never greater than 0.2 weeks and was statistically unstable (Table 5). Small, statistically unstable increases in the ORs for preterm delivery (*n* = 96) were also observed among PCE-exposed women (ORs = 1.1–1.9; data not shown). These increases were more pronounced when peak annual exposure before pregnancy (ORs = 1.2–1.7) and average monthly exposure during the LMP year (ORs = 1.4–1.7) were examined. The results of the multivariate analyses were unchanged when an indicator term for drinking-water source was included, and when certain variables (e.g., history of a preterm birth) were excluded.

No meaningful patterns of effect measure modification were observed according to maternal age, prior pregnancy losses, and tap and bottled water use (data not shown). However, when the data were stratified according to showering practices, mothers who took long showers (that is, > 70 min/week) at an exposed residence during the LMP year had heavier babies with longer gestations than mothers who did not take long showers at an exposed residence. The mean differences in birth weight and gestational duration were 103.4 g and 1.9 weeks, respectively. No effect modification was seen for showering temperature.

## Discussion

The results of this study suggest that prenatal PCE exposure, at the levels experienced by the study population, does not have an adverse impact on birth weight or gestational duration. Compared with unexposed children, the adjusted mean differences in birth weight were 20.9, 6.2, 30.1, and 15.2 for children whose mothers’ average monthly PCE exposure during the LMP year ranged from the lowest to highest quartile. Similarly, the adjusted mean differences in gestational age were −0.2, 0.1, −0.1, and −0.2 weeks for children whose mothers’ average monthly PCE exposure during the LMP year ranged from the lowest to highest quartile. The results were similar when cumulative and peak exposure before pregnancy were examined.

These results are likely affected by exposure misclassification. Because individual exposure measurements were unavailable, we estimated historical PCE exposures using a leaching and transport model (Webler and Brown 1993) that estimated the mass of PCE delivered to each residence. Nevertheless, results from a validation study indicate reasonable correlation between exposure estimates based on the Webler–Brown algorithm and PCE concentrations in historical water samples (Spearman correlation coefficient = 0.48, *p* < 0.0001; Spence LA, Aschengrau A, Gallagher L, Webster T, Heeren T, Ozonoff D, unpublished data).

The EPANET software estimated the amount of PCE that entered the home, so we do not know each subject’s precise exposure from showering, bathing, and tap-water consumption, which can result in dermal, oral, and inhalation exposure (Vieira et al. 2005). Although we obtained information on mothers’ water consumption and bathing habits before and during pregnancy, this information was difficult to recall accurately because it occurred so long ago. Thus, exposure misclassification is also likely from unmeasured or poorly measured physical and behavioral factors that influenced exposure. Poor recall also made it difficult to detect effect measure modification by drinking and bathing habits.

Exposure misclassification may also stem from our measures of prenatal PCE exposure, particularly cumulative exposure which combined both dose and duration. However, our analyses of peak prenatal exposure, which focused only on dose, did not find any adverse impacts on birth weight or gestational duration.

Even though exposure misclassification is probable, the preliminary results of an ongoing validation study suggest that its magnitude is modest, particularly given the quartile exposure categories used in the current analysis. Nevertheless, we cannot rule out a small increased risk of prenatal PCE exposure on the outcomes under study, given the likely downward bias of the misclassification. Last, it is possible that a null result was observed for birth weight and gestational duration because PCE exposure can lead to spontaneous abortion.

The current study has numerous strengths, including a relatively large sample size with a wide range of exposure levels, data on birth weight and gestational duration from birth certificates, and information on many confounding variables. In addition, levels of other measured drinking-water contaminants were low (Swartz et al. 2003). Trihalomethane levels were low because only one surface-water source in this region was treated with chlorination. Furthermore, the results were unchanged when drinking-water source (surface vs. ground) was controlled. Although nonparticipating mothers were younger, less educated, and had lighter babies than participating mothers, these differences were present for both exposed and unexposed nonparticipants, so it is unlikely they biased the current results.

Animal experiments suggest that an adverse effect on birth weight and size occurs in several species after prenatal exposure to PCE and the closely related solvent TCE (Bross et al. 1983; Dorfmueller et al. 1979; Elovaara et al. 1979; Healy et al. 1982; Saillenfant et al. 1995; Schwetz et al. 1975). Detrimental effects were seen only at high exposure levels in some experiments [e.g., 1,800 ppm TCE (Dorfmueller et al. 1979)] but were observed at relatively low levels in others [e.g., 1 μM TCE (Bross et al. 1983); 100 ppm TCE (Healy et al. 1982)].

Epidemiologic studies of women exposed occupationally to solvents including dry cleaning and degreasing agents have inconsistent results regarding an adverse effect on birth weight and gestational duration. Eight studies of birth weight found null results (Axelsson et al. 1984; Bosco et al. 1987; Hewitt and Tellier 1998; Laslo-Baker et al. 2004; McDonald et al. 1987; Olsen and Rachootin 1983; Seidler et al. 1999; Windham et al. 1991), whereas four studies observed 1.5- to 2.7-fold increases in the risk of low birth weight or moderate declines in mean birth weight (−82 to −168 g) (Ha et al. 2002; Hewitt and Tellier 1998; Khattak et al. 1999; Lipscomb et al. 1991). Three previous occupational studies found null associations between prenatal solvent exposure and gestational duration (Ha et al. 2002; Laslo-Baker et al. 2004; Savitz et al. 1996), whereas four studies found positive associations, ranging from 1.3- to 3.1-fold increases in the risk of preterm delivery (Hewitt and Tellier 1998; Khattak et al. 1999; Lipscomb et al. 1991; Savitz et al. 1989).

The occupational studies are difficult to compare with the present study because most workers were exposed to relatively high doses of a variety of solvents, solvent mixtures, and other noxious chemicals. Furthermore, data on important confounding factors such as cigarette smoking were often unavailable, and the numbers of exposed women were quite small, thereby reducing the ability of these studies to detect even a modest effect.

It is also difficult to generalize results seen among women exposed occupationally to solvents to those in the general population because of differences in socioeconomic status and reproductive history. For example, women who either cannot find work or do not have a monetary incentive to work are not represented in the occupational studies. In addition, women whose pregnancy history consists only of adverse outcomes such as spontaneous abortions are more likely to remain in the work force, whereas those who have had live-born children are more likely to drop out (Joffe 1985).

Prior community-based epidemiologic studies of solvent contaminated drinking water are more comparable to the current investigation. All prior drinking-water studies on PCE and TCE contamination had null findings for gestational duration (Bove et al. 1995; Massachusetts Department of Public Health 1996; Sonnenfeld et al. 2001). Although three prior studies observed no meaningful increases in the risk of low birth weight (Lagakos et al. 1986; Rodenbeck et al. 2000; Sonnenfeld et al. 2001), two studies observed increases in the risk of very low birth weight (defined as < 1,500 g) (Bove et al. 1995; Rodenbeck et al. 2000). In addition, some studies reported adverse effects in certain subgroups. These included an increased risk of growth retardation among women with moderate and high third-trimester solvent exposure (Massachusetts Department of Public Health 1996), and decreased birth weights among women ≥ 35 years of age (adjusted mean difference = −130 g; 90% CI, −236 to −23) and women with a history of two or more fetal losses (−104 g; 90% CI, −174 to −34) (Sonnenfeld et al. 2001). In our study, the occurrence of very low birth weight was too low to conduct meaningful analyses, and the exposure data were not detailed enough to conduct analyses by trimester. In addition, we did not observe effect measure modification by maternal age or a history of pregnancy losses.

Drinking-water studies with positive associations had water contaminant levels ranging from 14 to 215 μg/L PCE (Bove et al. 1995; Sonnenfeld et al. 2001), and from 55 to 107 μg/L TCE (Bove et al. 1995; Rodenbeck et al. 2000). In comparison, PCE levels in residential water supplies of Cape Cod in 1980 ranged from undetectable to 780 μg/L, with a mean of 45 μg/L (Spence LA, Aschengrau A, Gallagher L, Webster T, Heeren T, Ozonoff D, unpublished data). Thus, it is likely that some of our subjects had greater exposures than seen in the studies from New Jersey, North Carolina, and Arizona.

Demographic differences between our study population and that of several prior studies may account for the diverse findings. The other study populations had substantial proportions of women who were black or Hispanic, had low educational levels, and had inadequate levels of prenatal care. (Bove et al. 1995; Rodenbeck S, personal communication; Rodenbeck et al. 2000; Sonnenfeld et al. 2001). In contrast, as described in Table 2, mothers in our study were predominantly white, well educated, and connected to prenatal care. These characteristics are consistent with the low rates of low birth weight and very low birth weight seen in our population compared with rates in the general population (National Center for Health Statistics 2004), and may weaken the adverse effects of prenatal exposure to contaminated drinking water.

In summary, we found no association between prenatal PCE exposure and birth weight and gestational duration. Our results suggest that prenatal PCE exposure does not have an adverse effect on these outcome measures at the levels experienced by our study population. Given the likelihood of exposure misclassification, we cannot rule out a small adverse effect of PCE on these birth outcomes. Furthermore, because the occurrence of very low birth weight was very low in our population, this study cannot provide evidence for or against an association with this outcome. Last, it is possible that our results are not generalizable to more vulnerable populations. Because PCE remains a commercially ubiquitous solvent and common contaminant of drinking-water supplies (Agency for Toxic Substances and Disease Registry 1997; Moran et al. 2007), it is important to understand its effect on women and their pregnancies.

## Figures and Tables

**Table 1:** Selection, enrollment, and exposure status of study births, Cape Cod, Massachusetts, 1969–1983.

	Initial exposure status^a^		
	Exposed	Unexposed	Total
Selected (no.)	1,910	1,928	3,838
Excluded during enrollment (no.)			
Never located	161	147	308
No response	306	375	681
Ineligible	3	5	8
Refusal	200	151	351
Returned questionnaire (no.)	1,240	1,250	2,490
Percent of selected	64.9	64.8	64.9
Percent of located	70.9	70.2	70.5
Excluded during exposure assessment and analysis (no.)			
Inadequate residential history	73	96	169
Multiple birth	36	53	89
Congenital anomaly present	44	44	88
Missing last menstrual period	10	9	19
Available for analysis (no.)	1,077	1,048	2,125
Percent of selected	56.4	54.4	55.4

a After the more refined EPANET exposure assessment, the exposure designations of 496 children changed, so there were 1,353 exposed and 772 unexposed subjects in the final analysis.

**Table 2:** Distribution of selected characteristics of exposed^a^,^b^ and unexposed^b^ mothers [no. (%)].

Characteristic	Exposed	Unexposed	Characteristic	Exposed	Unexposed
Year of birth			Alcohol consumption during pregnancy		
1969–1973	136 (10.1)	100 (13.0)	Yes, during all trimesters	345 (25.5)	194 (25.1)
1974–1978	446 (33.0)	246 (31.9)	Yes, during some trimesters	176 (13.0)	107 (13.9)
1979–1983	771 (57.0)	426 (55.2)	No	799 (59.1)	459 (59.5)
Infant’s sex			Missing	33 (2.4)	12 (1.6)
Male	693 (51.2)	378 (49.0)	Diabetes before or during pregnancy		
Female	660 (48.8)	394 (51.0)	Yes	43 (3.2)	22 (2.8)
Age (mean ± SD)	1,353 (27.5 ± 4.5)	772 (27.6 ± 4.6)	No	1,283 (94.8)	734 (95.1)
Race			Missing	27 (2.0)	16 (2.1)
White	1,291 (95.4)	752 (97.4)	Hypertension or preeclampsia before or during pregnancy		
Nonwhite	62 (4.6)	20 (2.6)	Yes	96 (7.1)	63 (8.2)
Educational level			No	1,227 (90.7)	692 (89.6)
Less than high school	56 (4.1)	21 (2.7)	Missing	30 (2.2)	17 (2.2)
High school graduate	48 (35.5)	273 (35.4)	Bleeding, placental abruption, or previa during pregnancy		
Some college	404 (29.9)	253 (32.8)	Yes	126 (9.3)	69 (8.9)
Four year college graduate or higher	413 (30.5)	225 (29.1)	No	1,212 (89.6)	696 (90.2)
Parity			Missing	15 (1.1)	7 (0.9)
1	522 (38.6)	304 (39.4)	Viral infection during pregnancy		
2	479 (35.4)	282 (36.5)	Yes	15 (1.1)	8 (1.0)
≥ 3	336 (24.8)	178 (23.1)	No	1,323 (97.8)	757 (98.1)
Missing	16 (1.2)	8 (1.0)	Missing	15 (1.1)	7 (0.9)
Any prior pregnancy loss			Cervical incompetence during pregnancy		
Yes	219 (16.2)	119 (15.4)	Yes	24 (1.8)	18 (2.3)
No	1,118 (82.6)	645 (83.5)	No	1,314 (97.1)	747 (96.8)
Missing	16 (1.2)	8 (1.0)	Missing	15 (1.1)	7 (0.9)
Any prior low-birth-weight infant			History of infertility before pregnancy		
Yes	68 (5.0)	26 (3.4)	Yes	185 (13.7)	96 (12.4)
No	1,261 (93.2)	731 (94.7)	No	1,162 (85.9)	674 (87.3)
Missing	24 (1.8)	15 (1.9)	Missing	6 (0.4)	2 (0.3)
Any prior preterm delivery			Occupational exposure to solvents before or during pregnancy		
Yes	84 (6.2)	42 (5.4)	Yes, before or during	152 (11.2)	77 (10.0)
No	1,243 (91.9)	716 (92.7)	Yes, unknown when	24 (1.8)	8 (1.0)
Missing	26 (1.9)	14 (1.8)	No	1,146 (84.7)	666 (86.3)
Interpregnancy interval			Missing	31 (2.3)	21 (2.7)
First live birth	526 (38.9)	306 (39.6)	Residential proximity to dry cleaning establishment		
< 12 months	133 (9.8)	65 (8.4)	Yes	6 (0.4)	5 (0.6)
12–23 months	234 (17.3)	138 (17.9)	No	1,330 (98.3)	753 (97.5)
≥ 24 months	447 (33.0)	256 (33.2)	Missing	17 (1.3)	14 (1.8)
Missing	13 (1.0)	7 (0.9)	Use of solvent-based spot removers		
Adequacy of prenatal care during pregnancy			Occasionally or frequently	292 (21.6)	155 (20.1)
Adequate	989 (73.1)	555 (71.9)	Rarely	596 (44.1)	335 (43.4)
Intermediate	126 (9.3)	71 (9.2)	Never	440 (32.5)	262 (33.9)
Inadequate or no prenatal care	8 (0.6)	7 (0.9)	Missing	25 (1.8)	20 (2.6)
Missing	230 (17.0)	139 (18.0)	Use of professional dry cleaning		
Inadequate weight gain during pregnancy			Once per month or more	194 (14.3)	123 (15.9)
Yes	121 (8.9)	58 (7.5)	Less than once per month	764 (56.5)	405 (52.5)
No	1,178 (87.1)	680 (88.1)	Never	321 (23.7)	189 (24.5)
Missing	54 (4.0)	34 (4.4)	Missing	74 (5.5)	55 (7.1)
Cigarette smoking during pregnancy			Use of self-service dry cleaning		
Yes	354 (26.2)	219 (28.4)	Yes	126 (9.3)	95 (12.3)
No	973 (71.9)	539 (69.8)	No	1,190 (88.0)	659 (85.4)
Missing	26 (1.9)	14 (1.8)	Missing	37 (2.7)	18 (2.3)

a Ever exposed before birth.

b Based on final exposure designation.

**Table 3:** Distribution of cumulative PCE exposure before LMP, peak annual PCE exposure before LMP, and average monthly PCE exposure during LMP year among exposed subjects.

Cumulative	Cumulative exposure (g) before LMP month and year (*n* = 1,201)^a^	Peak annual exposure (g) before LMP year (*n* = 955)^a^	Average monthly exposure (g) during LMP year (*n* = 1,106)^a^
Minimum	2.8 × 10^−4^	1.212 × 10^−3^	1.176 × 10^−4^
10th percentile	1.1	1.3	3.98 × 10^−2^
5th percentile	5.6	5.6	0.2
Median	29.9	19.8	0.9
75th percentile	120.0	63.3	3.0
90th percentile	334.2	161.2	7.9
Maximum	3,904.2	1,770.7	147.6

a There were 860 individuals who were exposed both before and during the LMP year. Another 95 individuals were exposed before the LMP year but were unexposed during the LMP year, and another 246 individuals were exposed during the LMP year but were unexposed before the LMP year. Thus, cumulative exposure before the LMP month and year includes 1,201 exposed individuals (860 + 95 + 246), peak annual exposure before the LMP year includes 955 exposed individuals (860 + 95), and average monthly exposure during the LMP year includes 1,106 exposed individuals (860 + 246).

**Table 4:** Number of births and crude mean ± SD for birth weight and gestational duration by PCE exposure category.

	No.	Mean ± SD
Birth weight (g)		
Cumulative PCE exposure before LMP month and year		
≥ 75th	301	3,482 ± 516
50th– < 75th	300	3,511 ± 557
25th– < 50th	300	3,455 ± 486
> 0– < 25th	300	3,509 ± 500
0 (referent)	924	3,474 ± 490
Peak annual PCE exposure before LMP year		
≥ 75th	239	3,473 ± 523
50th– < 75th	239	3,533 ± 551
25th– < 50th	239	3,453 ± 479
> 0– < 25th	238	3,527 ± 503
0 (referent)	1,170	3,472 ± 495
Average monthly PCE exposure during LMP year		
≥ 75th	277	3,485 ± 495
50th– < 75th	276	3,483 ± 521
25th– < 50th	279	3,487 ± 570
> 0– < 25th	274	3,481 ± 481
0 (referent)	1,019	3,481 ± 490
Gestational duration (weeks)		
Cumulative PCE exposure before LMP month and year		
≥ 75th	301	40.1 ± 2.1
50th– < 75th	300	40.1 ± 2.5
25th– < 50th	300	40.2 ± 2.1
> 0– < 25th	300	40.2 ± 2.2
0 (referent)	924	40.2 ± 2.0
Peak annual PCE exposure before LMP year		
≥ 75th	239	40.1 ± 2.2
50th– < 75th	239	40.1 ± 2.5
25th– < 50th	239	40.2 ± 2.0
> 0– < 25th	238	40.0 ± 2.1
0 (referent)	1,170	40.2 ± 2.0
Average monthly PCE exposure during LMP year		
≥ 75th	277	40.1 ± 1.9
50th– < 75th	276	40.1 ± 2.4
25th– < 50th	279	40.3 ± 2.4
> 0– < 25th	274	40.0 ± 2.2
0 (referent)	1,019	40.2 ± 1.9

25th, 50th, and 75th are percentiles.

**Table 5:** Multivariate^a^ GEE analysis of birth weight and gestational duration by PCE exposure category.

	No.	β	SD	95% CI
Birth weight (g)				
Cumulative PCE exposure before LMP month and year				
≥ 75th	281	28.5	33.6	−37.4 to 94.4
50th– < 75th	279	53.7	34.3	−13.6 to 120.9
25th– < 50th	285	−5.4	29.3	−62.8 to 52.0
> 0– < 25th	279	42.1	31.8	−20.3 to 104.5
0 (referent)	867			
Peak annual PCE exposure before LMP year				
≥ 75th	224	14.6	36.0	−55.9 to 85.1
50th– < 75th	220	65.1	33.7	−1.0 to 131.2
25th– < 50th	228	17.9	31.7	−44.2 to 80.0
> 0– < 25th	230	70.2	34.9	1.8 to 138.7
0 (referent)	1,089			
Average monthly PCE exposure during LMP year				
≥ 75th	254	15.2	32.7	−48.9 to 79.3
50th– < 75th	257	30.1	33.3	−35.2 to 95.4
25th– < 50th	260	6.2	34.2	−60.8 to 73.3
> 0– < 25th	262	20.9	31.7	−41.2 to 82.9
0 (referent)	958			
Gestational duration (weeks)				
Cumulative PCE exposure before LMP month and year				
≥ 75th	287	−0.1	0.1	−0.4 to 0.1
50th– < 75th	279	−0.2	0.2	−0.5 to 0.2
25th– < 50th	284	−3.3E-02	0.1	−0.3 to 0.2
> 0– < 25th	282	−0.1	0.1	−0.4 to 0.2
0 (referent)	872			
Peak annual PCE exposure before LMP year				
≥ 75th	227	−0.1	0.1	−0.4 to 0.2
50th– < 75th	224	−0.2	0.2	−0.5 to 0.2
25th– < 50th	227	0.1	0.2	−0.2 to 0.4
> 0– < 25th	230	−0.2	0.2	−0.5 to 0.1
0 (referent)	1,096			
Average monthly PCE exposure during LMP year				
≥ 75th	259	−0.2	0.1	−0.4 to 0.1
50th– < 75th	256	−0.1	0.2	−0.5 to 0.2
25th– < 50th	261	0.1	0.2	−0.2 to 0.4
> 0– < 25th	265	−0.2	0.1	−0.5 to 0.1
0 (referent)	963			

25th, 50th, and 75th are percentiles.

a Birth weight adjusted for gestational age, maternal race, educational level, history of a low-birth-weight child, occupational exposure to solvents, use of self-service dry cleaning, and proximity of any residences to dry cleaning establishments. Gestational age adjusted for maternal race, educational level, history of a preterm delivery; complications such as placenta previa, placental abruption, and cervical incompetence; occupational exposure to solvents, use of self-service dry cleaning, and the proximity of any residences to dry-cleaning establishments.
